# Pharmacogenetic testing and monitoring of complete blood counts among Veterans newly prescribed thiopurine treatments: a retrospective cohort study

**DOI:** 10.1186/s40545-023-00657-7

**Published:** 2023-12-11

**Authors:** Nai-Chung Nelson Chang, Catherine Chanfreau-Coffinier, Jill Bates, Sony Tuteja, Tori R. Anglin, Von R. Moore, Jason Hou, Akbar Waljee, Kathryn M. Pridgen, David W. Oslin, Deepak Voora, Scott L. DuVall, Francesca E. Cunningham, Julie A. Lynch

**Affiliations:** 1 Department of Veterans Affairs Salt, Lake City Health Care System, 500 Foothill Drive, Salt Lake City, UT 84148 USA; 2 Durham VA Medical Center, Durham, NC USA; 3 Division of Practice Advancement and Clinical Education, UNC Eshelman School of Pharmacy, University of North Carolina at Chapel Hill, Chapel Hill, NC USA; 4 Perelman School of Medicine, University of Pennsylvania, Philadelphia, PA USA; 5 Corporal Michael Crescenz Department of Veterans Affairs Medical Center, Philadelphia, PA USA; 6 Department of Veterans Affairs Center for Medication Safety/Pharmacy Benefits Management Services, Hines, IL USA; 7 Center for Innovations in Quality, Effectiveness and Safety (IQuEST), Michael E. DeBakey Veterans Affairs Medical Center, Houston, TX USA; 8 Section of Gastroenterology and Hepatology, Baylor College of Medicine, Houston, TX USA; 9 Health Services Research and Development Center of Clinical Management Research, VA Ann Arbor, Ann Arbor, MI USA; 10 Michigan Integrated Center for Health Analytics and Medical Prediction (MICHAMP), Ann Arbor, MI USA; 11 Division of Gastroenterology and Hepatology, Department of Internal Medicine, Michigan Medicine, Ann Arbor, MI USA; 12 Duke Center for Applied Genomics and Precision Medicine, Department of Medicine, Duke University School of Medicine, Durham, NC USA; 13 Division of Epidemiology, Department of Internal Medicine, University of Utah School of Medicine, Salt Lake City, UT USA

## Abstract

Pharmacogenetic (PGx) testing before initiation of thiopurine treatment and CBC monitoring post-initiation helps avoid adverse events and ensure patient safety. This study aims to evaluate trends in PGx testing and CBC monitoring among Veterans prescribed azathioprine, thioguanine, or mercaptopurine to demonstrate VA’s efforts to improve medication safety after an adverse event. To assess testing patterns, we used VA electronic health report data to identify 20,524 Veterans who first began thiopurine treatment between January 1, 2010, to December 31, 2021. Aggregate monthly counts of thiopurine prescriptions and associated lab tests were tabulated, and the trend in the proportion of patients tested was analyzed using the Mann–Kendall test. The proportion of patients undergoing PGx testing rose from 30.0% in 2010 to 47.5% in late 2014 (July–December). However, PGx testing and overall testing only increased slightly after the sentinel event, and orders levelled off over time at slightly lower levels than before the sentinel event. Very little change was seen in the overall proportion of individuals receiving any testing across all patients with new prescriptions from the time of the sentinel event in 2014 to the end of 2021. A large portion of patients prescribed thiopurine drugs did not receive testing that could help prevent the development of potential adverse events, leading to a predominantly reactive approach. Increased PGx testing may result in a more proactive approach to the prevention of adverse events due to genetic interaction.

## Introduction

Patients with decreased activity in thiopurine *S*-methyl transferase (TPMT) have increased risk of toxic side effects from thiopurine drugs (i.e., azathioprine, thioguanine, and mercaptopurine) [1]. The FDA recommends that prescribers consider genetic or activity testing for TPMT deficiency prior to initiation of azathioprine treatment and monitor treatment using complete blood counts (CBCs) [2, 3]. *TPMT* genotype testing identifies patients as either normal metabolizers (two functional *TPMT* alleles), intermediate metabolizers (generally one non-functional allele), or poor metabolizers (two non-functional alleles). Reduced dose and alternative treatment may be considered for those at higher risk of developing myelotoxicity.

Intermediate or poor metabolizer status has been associated with a higher likelihood of azathioprine discontinuation due to myelotoxicity (HR = 2.90), and an analysis of patients with a *TPMT* variant showed that a thiopurine dose regimen based on pretreatment genotyping significantly decreased likelihood of hematologic adverse drug reaction [4, 5]. Additionally, a recent study found the occurrence of myelotoxicity was significantly lower in patients who had been genotyped prior to treatment than those retrospectively genotyped (2.0% vs. 21.2%, *p* < 0.001) [6].

In October 2014, a Veteran at the Department of Veterans Affairs (VA) died from a potential adverse reaction to azathioprine [7]. Gaps in testing for patients prescribed thiopurines and a lack of reliable documentation of pharmacogenetic (PGx) tests within the VA electronic health records (EHRs) were identified during a root cause analysis. Similar gaps in monitoring patients for drug-induced myelosuppression had been documented by VA clinicians studying inflammatory bowel disease in 2012 [8]. In response, VA National Pharmacy Benefit Management Office’s Center for Medication Safety (VAMedSAFE) published monitoring recommendations and launched a risk-reduction initiative using their Medication Use Evaluation Tracker (MUET) program in 2019 [9]. This clinical dashboard identifies patients prescribed azathioprine or mercaptopurine and evaluates whether patients are being monitored in accordance with manufacturer recommendations and practice guidelines.

We tested our hypothesis that the sentinel event and the VA’s response effected lasting change in CBC and PGx testing patterns among Veterans starting thiopurine therapy. A secondary objective was identifying the rate of TPMT testing by prescriber specialty.

## Methods

For this retrospective cohort study, data were analyzed as part of a study protocol approved by VA Institutional Review Board.

We analyzed medical record data from the VA Corporate Data Warehouse (CDW). Using outpatient visits and pharmacy records, we identified Veterans who began their first thiopurine treatment between January 1, 2010, and December 31, 2021. We collected data on TPMT genetic and enzyme activity tests from the VA Genetic and Molecular Diagnostic Test Database (VA GDx) and extracted CBC test orders from the CDW. Test results were curated to ensure standardization. Although *NUDT15* PGx testing is also relevant, it was excluded because fewer than 11 patients were tested.

The primary outcome of interest was the testing status of a patient. Testing category was assigned using a hierarchical approach. Patients undergoing PGx testing were classified as tested/monitored; remaining patients were classified as either CBC tested or not tested.

We excluded inpatient populations due to a high likelihood of CBC testing for reasons other than thiopurine monitoring. We used proxies to tie prescriptions to specific settings and applications that may present different guidelines on testing. We identified the medical specialty of the ordering provider using stop codes and CDW outpatient data.

Over the observation period, we tabulated the aggregate monthly counts of thiopurines prescriptions and the counts of the TPMT associated laboratory tests that were ordered to determine the percentage of patients undergoing PGx testing at any time before prescription or PGx and CBC testing within 30 days after prescription. Using multiple logistic regression, we determined the likelihood of a patient being tested receiving PGx TPMT testing before prescription (genetic and enzyme activity tests only) or after prescription (all tests). All statistical analyses were conducted using R version 4.1.2 (Vienna, Austria). To determine the trend in testing, we used the Mann–Kendall test to analyze the proportion of patients tested between 2010–2022 and identified the slope using the Theil-Sen estimator.

We conducted a sensitivity analysis to confirm that expanding the period to 60 or 90 days following treatment initiation generated equivalent results. We also examined the healthcare utilization of untested patients and determined that lack of testing was not due to lack of care access at VA.

## Results

**Fig. 1:**
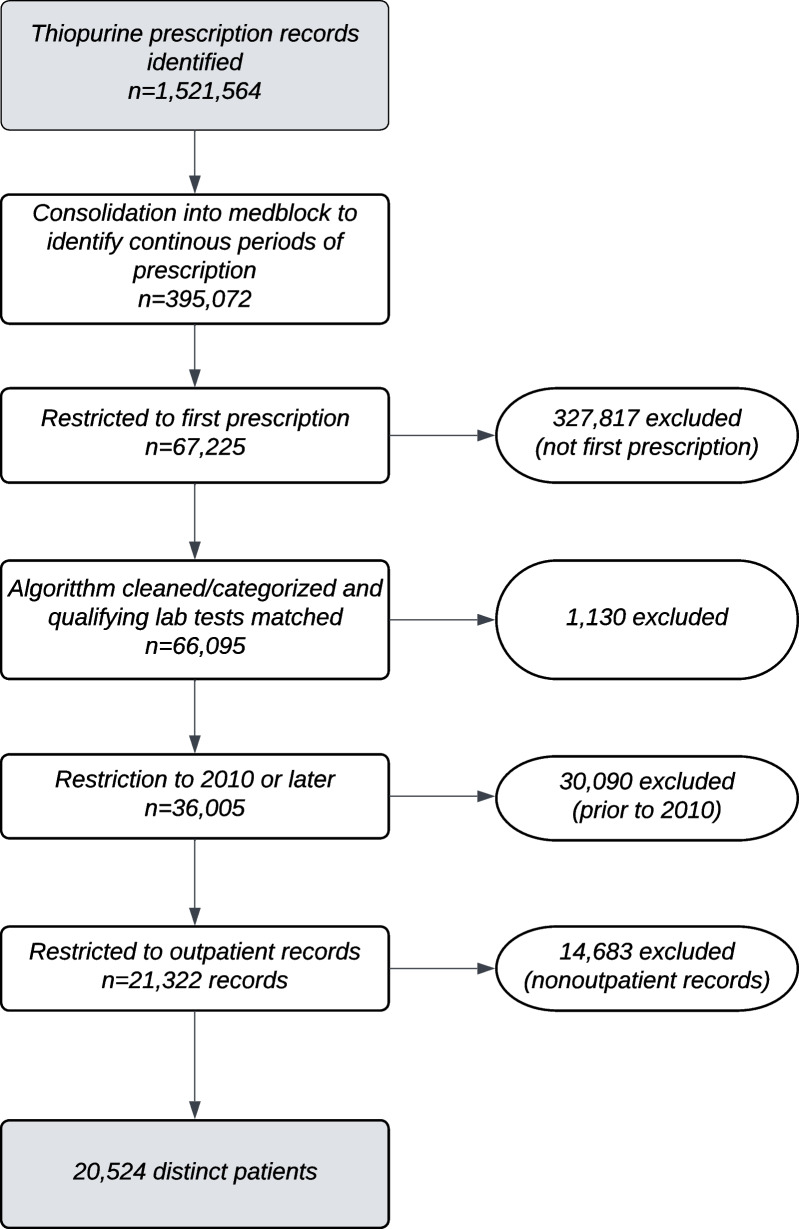
Cohort selection

We identified 20,524 patients who met our inclusion criteria (Fig. 1). Among these patients, 17,655 were prescribed azathioprine (86.0%), 3245 (15.8%) were prescribed mercaptopurine, and 47 (0.2%) were prescribed thioguanine. Most patients underwent either PGx or CBC tests (*n* = 13,535; 65.9%), and most tested patients received PGx tests (*n* = 9064; 67.0% of all tested patients) (Table 1).

**Table 1:** Demographics of Veterans newly prescribed thiopurine treatment

Variable	All		Tested^a^		Not tested^a^		*p*-value
	Number	Percent	Number	Percent	Number	Percent	
Total	20,524	–	13,535	65.9	7060	34.4	
Sex							< 0.05
Female	2748	13.4	1950	14.4	809	11.5	
Male	17,776	86.6	11,585	85.6	6251	88.5	
Race^b^							< 0.05
Asian	159	0.8	125	0.9	34	0.5	
American Indian or Alaska Native	136	0.7	90	0.7	46	0.7	
Black or African American	3670	17.9	2659	19.6	1019	14.4	
Native Hawaiian or Other Pacific Islander	167	0.8	102	0.8	66	0.9	
White	15,104	73.6	9763	72.1	5398	76.5	
Unknown	1288	6.3	796	5.9	497	7.0	
Ethnicity^b^							< 0.05
Hispanic or Latino	1113	5.4	796	5.9	323	4.6	
Not Hispanic or Latino	18,815	91.7	12,425	91.8	6455	91.4	
Unknown	596	2.9	314	2.3	282	4.0	
Drug type							< 0.05
Azathioprine	17,655	86.0	11,551	85.3	6104	86.5	
Mercaptopurine	3245	15.8	2268	16.8	977	13.8	
Thioguanine	47	0.2	22	0.2	25	0.4	
Prescriber specialty							< 0.05
Dermatology	659	3.2	541	4.0	118	1.7	
Gastroenterology	6666	32.5	5652	41.8	1051	14.9	
Hematology	104	0.5	78	0.6	26	0.4	
Oncology	117	0.6	87	0.6	30	0.4	
Rheumatology	3821	18.6	3019	22.3	802	11.4	
Transplant	30	0.1	22	0.2	8	0.1	
Other	9235	45.0	4203	31.1	5038	71.4	
Test type							–
Genetic or activity	9064	44.2	9064	67.0	–	–	
Blood	4489	21.9	4489	33.2	–	–	
Not tested	7060	34.4	–	–	7060	100.0	

^a^Tested (i.e., with any genetic or activity tests for TPMT or CBC) and Not Tested have slight overlap due to order of medication given

^b^Race and ethnicity information about patients was self-reported and taken from the EHR

The adjusted regression model analyzing the likelihood of patients undergoing either PGx testing or monitoring by CBC identified statistically significant differences by medication, patient race, age, specialty provider, and setting. (Table 2) In both models, those prescribed mercaptopurine and thioguanine were less likely to be tested and Black and Asian patients were more likely to be tested than White patients. The likelihood of being tested was also significantly greater for patients treated by dermatology and gastroenterology compared to those treated by other specialties in both models.

**Table 2:** Likelihood of receiving testing

	Tested by CBC or PGx		PGx testing only	
	O.R. (95% CI)	*p* value	O.R. (95% CI)	*p* value
Drug type				
Azathioprine	Ref.		Ref.	
Mercaptopurine	0.84 (0.80–0.88)	< 0.05	0.95 (0.90–0.99)	< 0.05
Thioguanine	0.56 (0.41–0.76)	< 0.05	0.39 (0.26–0.59)	< 0.05
Prescribing Dx or stop code				
Dermatology	Ref.		Ref.	
Gastroenterology	1.13 (1.02–1.26)	0.25	0.91 (0.83–0.99)	0.26
Hematology	0.71 (0.55–0.91)	< 0.05	0.05 (0.04–0.07)	< 0.05
Oncology	0.65 (0.52–0.82)	0.07	0.05 (0.04–0.07)	< 0.05
Rheumatology	0.19 (0.17–0.21)	< 0.05	0.12 (0.11–0.13)	< 0.05
Transplant	0.76 (0.69–0.85)	0.01	0.55 (0.51–0.61)	< 0.04
Other	0.60 (0.39–0.91)	0.23	0.07 (0.04–0.13)	< 0.05
Age	0.99 (0.99–0.99)	< 0.05	0.99 (0.99–0.99)	< 0.05
Sex				
Male	Ref.		Ref.	
Female	0.95 (0.90–1.00)	0.28	1.00 (0.95–1.04)	0.92
Race^a^				
White	Ref.		Ref.	
Black or African American	1.30 (1.24–1.36)	< 0.05	1.24 (1.19–1.30)	< 0.05
Asian	1.74 (1.42–2.14)	< 0.06	1.41 (1.18–1.69)	< 0.05
Native Hawaiian or Other Pacific Islander	0.77 (0.65–0.91)	0.13	0.89 (0.75–1.06)	0.50
American Indian or Alaska Native	1.16 (0.95–1.40)	0.45	1.09 (0.90–1.32)	0.65
Unknown	0.97 (0.91–1.04)	0.70	1.00 (0.93–1.07)	1.00
Ethnicity^a^				
Not Hispanic or Latino	Ref.		Ref.	
Hispanic or Latino	1.16 (1.08–1.25)	< 0.05	1.08 (1.01–1.16)	0.27
Unknown	0.69 (0.63–0.76)	< 0.05	0.84 (0.76–0.93)	< 0.05

^a^Race and ethnicity information about patients was self-reported and taken from the EHR

**Fig. 2:**
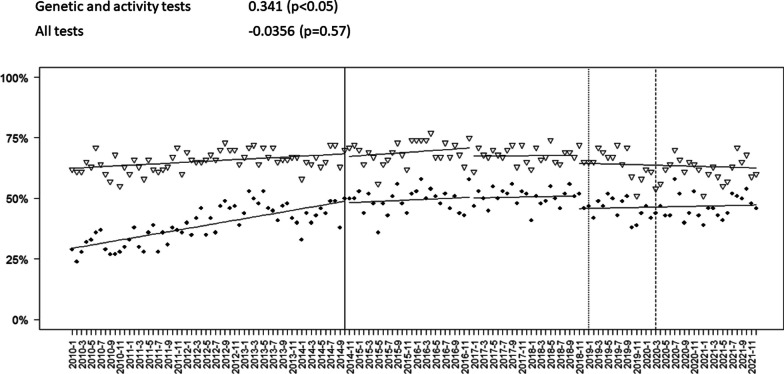
Overall trend using Mann–Kendall test

The rate of PGx testing changed significantly over time (Mann–Kendall Tau: 0.339, *p* < 0.05; Sen’s slope: 0.09), but the rate of overall testing did not (Mann–Kendall Tau: − 0.0312, *p* = 0.57). (Fig. 2) The proportion of patients undergoing PGx testing rose from 30.0% in 2010 to 47.5% in late 2014 (July–December). However, PGx testing and overall testing only increased slightly after the sentinel event, and orders levelled off at slightly lower levels than before the sentinel event. From October 2014 to December 2021, minimal change occurred in the overall proportion of individuals receiving any testing across all patients with new prescriptions, including immediately following the launch of the MUET program.

## Discussion

We found that testing rates did not significantly increase following the sentinel event and that testing rates varied significantly depending on ordering specialty. These findings suggest that significant opportunities for improvement in testing continue to exist.

The variation in testing rates among specialties may be due to varying treatment norms. Specialties with lower testing rates included hematology, oncology, and transplant medicine, where patients are likely to undergo routine blood tests during treatment, ensuring up-to-date status regarding any potential adverse events. In specialties with significantly more PGx testing (e.g., dermatology, gastroenterology, and rheumatology), azathioprine is prescribed to combat inflammation, and most prescriptions are for outpatients, which may result in fewer opportunities for CBC monitoring.

Race-related differences in testing rates may be related to provider awareness of racial differences in TPMT genotype and phenotype status. Studies show lower TPMT activity in Black individuals than in White or Asian individuals, and Dickson et al. observed that Black patients discontinued azathioprine due to hematopoietic toxicity at a higher rate than White patients [4, 10–12]. Additionally, studies indicate that the prevalence of TPMT variants differs among races; the most common variant allele is TPMT*3A in White individuals and TPMT*3C in Black and Asian individuals [13]. Research has observed a lower prevalence of TPMT variants among Chinese and South Asian individuals than White individuals [14].

The initial increase in PGx testing from 2010 to 2014 was likely due to an increased understanding and availability of TPMT PGx testing; for instance, the initial CPIC^®^ guideline for TPMT testing and dosing was published in 2011 [15]. A similar pattern of increased testing was observed by Dickson et al. although in that patient population, testing continued to increase through 2018 [4]. Within our cohort, a significant portion of patients prescribed thiopurines were still not tested either before or after treatment initiation. Additionally, neither the sentinel event nor implementation of MUET was associated with a significant increase in testing.

We believe that the current, predominantly reactive, paradigm for treating patients with potentially decreased TPMT activity is insufficient. Variation in practices among various specialties presents significant challenges to the implementation of TPMT PGx testing and dissemination of testing results [16]. Additionally, as shown by Coenen et al., adoption of one form of testing does not exclude or trivialize other tests, and continued monitoring by CBCs even after TPMT PGx testing resulted in fewer patients with hematological toxicity [5]. Thus, integrating information on testing status and results in the EHRs is critical to ensure that providers are aware of testing needs and available genetic information to guide therapeutic decisions. A combination of increased PGx testing and the incorporation of decision supports and clinical dashboards into EHR systems may result in a more proactive approach to the prevention of drug-use adverse events associated with genetic variants.

This study was limited by constraints of the VA population. Although the VA is the largest healthcare system in the U.S., its population is predominantly male and older. We determined the likely sources of the prescription for inpatient population based on chronological proximity and not direct link to the prescription. To limit potential bias from past exposure to thiopurines, we only included patients who were newly initiated on the treatment. We did not report TPMT testing performed independent of thiopurine treatment initiation.

## Declarations

### Availability of data and materials

Patient-level data are already accessible to all VA researchers with appropriate IRB approvals. Access for researchers who are not VA-credentialed is being pilot-tested on VA Data Commons (data-commons.org). Data is available for any type of analysis.
